# Magnetic field induced motion behavior of gas bubbles in liquid

**DOI:** 10.1038/srep21068

**Published:** 2016-02-12

**Authors:** Keliang Wang, Pucheng Pei, Yu Pei, Ze Ma, Huachi Xu, Dongfang Chen

**Affiliations:** 1 State Key Lab. of Automotive Safety and Energy, Tsinghua University, Beijing 100084, China; 2 Collaborative Innovation Center of Intelligent New Energy Vehicle, China

## Abstract

The oxygen evolution reaction generally exists in electrochemical reactions. It is a ubiquitous problem about how to control the motion of oxygen bubbles released by the reaction. Here we show that oxygen bubbles during oxygen evolution reaction exhibit a variety of movement patterns in the magnetic field, including directional migration and rotational motion of oxygen bubbles when the magnet in parallel with the electrode, and exclusion movement of oxygen bubbles when the magnet perpendicular to the electrode. The results demonstrate that the direction of oxygen bubbles movement is dependent upon the magnet pole near the electrode, and the kinetics of oxygen bubbles is mainly proportional to intensity of the electromagnetic field. The magnetic-field induced rotational motion of oxygen bubbles in a square electrolyzer can increase liquid hydrodynamics, thus solve the problems of oxygen bubbles coalescence, and uneven distribution of electrolyte composition and temperature. These types of oxygen bubbles movement will not only improve energy saving and metal deposition for energy storage and metal refinery, but also propel object motion in application to medical and martial fields.

The oxygen evolution reaction is widely involved in rechargeable metal-air batteries, regenerative fuel cells, electrochemical water-splitting, electrodeposition, electro-refining and electro-synthesis^1^,^2^,^3^,^4^,^5^,^6^,^7^. It is well-known that oxygen bubbles adhered to the electrode surface would block the following electrochemical reaction^8^,^9^, and the sluggish kinetics of oxygen evolution reaction is mostly dependent upon the activity of catalysts^10^,^11^. However, gas bubbles motion in a controlled way is still a challenge. To solve the problem of object propulsion, an external field is generally employed, including electric field, magnetic field, and ultraviolet light. Loget *et al.*^12^,^13^ discovered that locomotion of conducting objects can be controlled by bubble propulsion in the bipolar electrochemistry. Chang *et al.*^14^ demonstrated that semiconductor diodes suspended in water may be propelled by an external electric field. Dreyfus *et al.*^15^ found that the motion of a flexible magnetic filament could be actuated by an external magnetic field. Ghosh *et al.*^16^ reported that the operation of chiral colloidal propellers in water can be navigated with micron-level precision by means of homogeneous magnetic fields. Ibele *et al.*^17^ stated that silver chloride particles in deionized water would exhibit a schooling behavior when exposed to ultraviolet light. Ismagilov *et al.*^18^ proposed that hemicylindrical plates can move under the impulse of bubbles generated by the platinum-catalyzed decomposition of hydrogen peroxide. In addition, object motion can also be driven by the filament rigidity and hydrodynamic friction^19^. Therefore, Object motion can be propelled by means of either energy self-transformation or external propulsion. Species transport in the fluid may be controlled by diffusion, migration, and convection, where diffusion derives from concentration difference, migration from additional power, and convection from fluid dynamics. In addition, Oxygen molecules were found to possess paramagnetic characteristics due to unpaired electrons in its molecular structure^20^,^21^.

In this work, we investigated the effect of the magnetic field on oxygen bubbles movement during oxygen evolution reaction, where a cross made of carbon fiber was used for quantitative description of oxygen bubbles movement at different magnetic induction intensities, electric field intensities and temperatures.

## Results

### Magnetic field induced linear motion of oxygen bubbles

A set of experiments was carried out under the condition of bipolar electrochemistry. The electrochemical reactions on both surfaces of the anode and cathode are as follows:


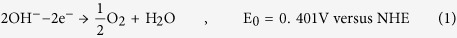



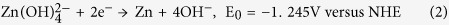


**Figure 1:**
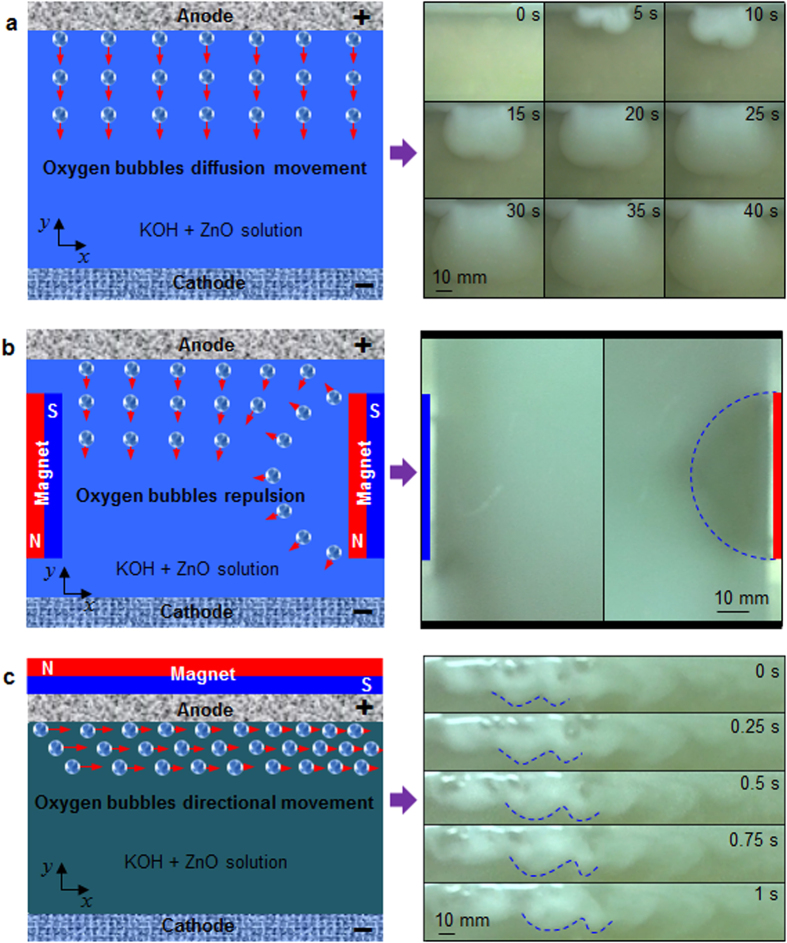
Locomotion of oxygen bubbles in the upright elongated cell. (**a**) Oxygen bubbles movement perpendicular to the anode surface during oxygen evolution reaction without magnetic field. (**b**) Oxygen bubbles away from the magnet when the magnet N-pole perpendicular to the electrode. (**c**) Oxygen bubbles movement parallel to the anode surface during oxygen evolution reaction when the magnet S-pole faced with the anode.

The direction of oxygen bubbles movement is perpendicular to the anode surface due to oxygen concentration gradient in the neighborhood of the anode, as shown in Fig. 1a. Once oxygen concentration in the electrolyte near the anode gets saturated, most of oxygen bubbles will spread away from the anode surface, whereas part of oxygen bubbles adhered to the anode surface would increase the charging voltage, leading to morphological change of electrodeposited zinc^22^,^23^,^24^. However, Oxygen bubbles movement happens to change in the case of the magnet close to the electrode. If the magnet is perpendicular to the electrode, as shown in Fig. 1b, oxygen bubbles will get together near the magnet in the case of the magnet S-pole faced with the electrolyte, while oxygen bubbles would be far away from the magnet under the condition of the magnet N-pole faced with the electrolyte. When the magnet S-pole is faced with the anode, oxygen bubbles will orderly move towards the right, as shown in Fig. 1c. The moving direction of oxygen bubbles is not perpendicular but approximately parallel to the anode surface. When the magnetic pole alters, namely the magnet N-pole is confronted with the anode, oxygen bubbles will move towards the left. In addition, oxygen bubbles between the anode and the cathode are quickly carried away by way of the magnetic field, reducing the ohmic resistance. This means that the direction of oxygen bubbles movement can be controlled by the magnet pole near the electrode. In fact, the above phenomena can also occur when a magnet is faced with the cathode, but oxygen bubbles just move the opposite direction. Two magnets with opposite poles are located in the anode and cathode, respectively, which can strengthen oxygen bubbles movement.

**Figure 2:**
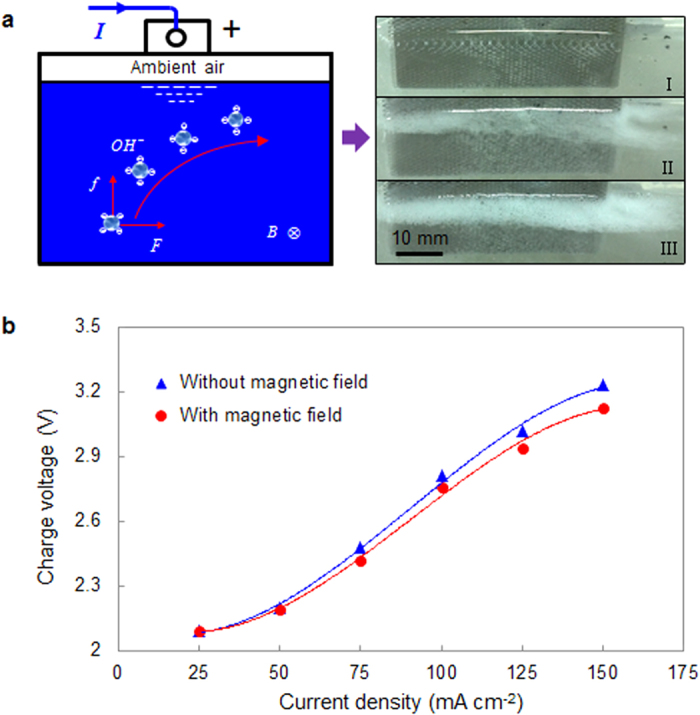
Analysis of oxygen bubbles movement. (**a**) The effect of Lorentz force *F* and buoyance *f* on the oxygen bubbles movement. (**b**) Charging voltage contrast at different current densities with /without magnetic field.

It is well-known that hydroxyl ions aggregate near the anode on account of the polarization effect. Oxygen bubbles move upward at the buoyancy force, thus anions adjacent to bubbles will be accompanied with the motion of oxygen bubbles, resulting in the formation of ion current. Once the magnetic field applied is perpendicular to the bubbles motion, Hull effect will happen. Therefore, oxygen bubbles in the rising process are subjected to the Lorenz force in horizontal direction, leading to tilted upward movement of oxygen bubbles, as shown in Fig. 2a. When getting to the electrolyte surface, these oxygen bubbles will move along the electrode surface. Figure 2b shows the effect of the magnetic field on the charging performance of electrodeposited zinc, where the magnetic field is found to effectively reduce the charging voltage, and the effect of magnetic field on energy saving is more obvious with increase of current densities. The magnetic field can not only achieve directional migration of oxygen bubbles but also accelerate motion of oxygen bubbles.

### Magnetic field induced rotational motion of oxygen bubbles

**Figure 3:**
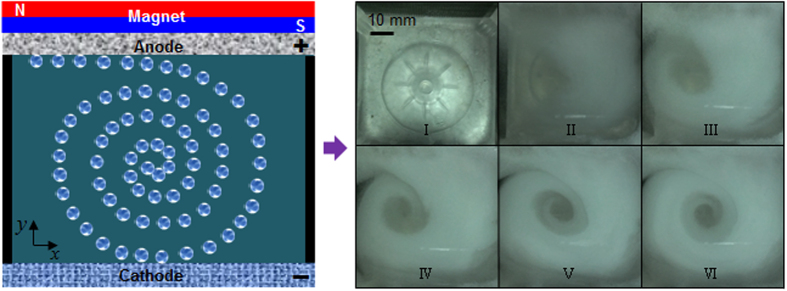
Schematic representation of oxygen bubbles movement in the upright square electrolyzer. Oxygen bubbles in clockwise motion during oxygen evolution reaction when the magnet S-pole confronted with the anode.

Provided that the experiment is carried out in a square electrolyzer, oxygen bubbles will be in rotational motion. When the magnet S-pole is faced with the anode, oxygen bubbles would be in clockwise motion in the electrolyte, as shown in Fig. 3. The electrolyte is propelled by oxygen bubbles movement, leading to the electrolyte whirlpool. If the magnet N-pole is confronted with the anode, oxygen bubbles will be in counterclockwise rotation. Rotational motion of oxygen bubbles in the electromagnetic field can increase electrolyte hydrodynamics, serving as a stirrer. It is helpful to enhance surface quality of electrodeposited zinc such as inhibition of dendrite growth of metal-air batteries during charging. In addition, hydrodynamic electrolyte can make oxygen bubbles detached from the anode surface, decreasing the internal resistance.

### Quantitative analysis of magnetic field induced oxygen bubbles movement

**Figure 4:**
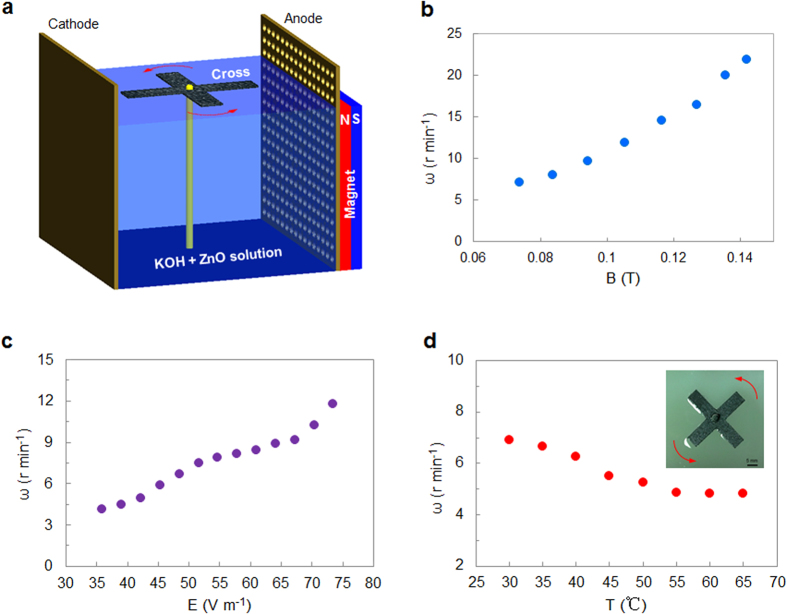
Influencing factors of oxygen bubbles kinetics in the electromagnetic field. (**a**) Schematic view of a horizontal rotor driven by oxygen bubbles. (**b**) Plot of the rotating speed ω of the rotor as a function of magnetic induction density B under the same electric field condition. (**c**) Plot of the speed ω of the rotor as a function of the electric field E under the same magnetic field condition. (**d**) The effect of electrolyte temperature T on the speed ω of the rotor under the same electromagnetic field condition

To probe into oxygen bubbles movement, a horizontal rotor was designed as shown in Fig. 4a, where the rotor is composed by a cross made of carbon fiber floating in the electrolyte and a tin wire axis fixed at the bottom of the electrolytic cell in order to eliminate the interference effect of the magnetic field. We investigated the effect of the magnetic field on the cross movement by means of altering the distance between the magnet and the anode, demonstrating that the rotating speed of the cross increases with increase of magnetic induction density (Fig. 4b). When the electric field intensity is enhanced (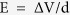, 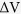 the applied voltage and 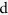 the distance between the anode and the cathode), much more oxygen bubbles will be produced during oxygen evolution reaction. The driving energy of the bubble itself would be strengthened, leading to the cross in rotation more quickly. Therefore, the rotating speed of the cross increases with the electric field (Fig. 4c). When electrolyte temperature rises, on the one hand, molecular Brownian movement gets enhanced, thus reducing the effect of molecular paramagnetism according to Curie’s law; on the other hand, ohmic resistance of the electrolyte will decrease at high temperatures, resulting in reduction of the electric field. As a consequence, the rotating speed of the cross decreases with the temperature (Fig. 4d). It can be found that oxygen bubbles movement can be controlled by the magnetic induction intensity and the electric field, but the temperature has a negative effect on the controlled motion of oxygen bubbles.

## Discussion

In view of the above mentioned, directional motion of oxygen bubbles during oxygen evolution reaction can be controlled by the magnetic field. Oxygen bubbles will move along the anode surface if there is no obstacle at the forward direction. Otherwise, oxygen bubbles would move rotationally. A cross of diamagnetic material was employed as experimental setup, demonstrating that object motion is not caused by electrophoresis or electro-osmosis but determined by bubble propulsion. Object motion is mainly related to the electric field, the magnetic induction intensity and electrolyte temperature, where the object speed is proportional to intensity of the electromagnetic field, but it decreases with electrolyte temperature. The magnetic field induced motion of oxygen bubbles may not only lead to the liquid whirlpool, but might also result in the liquid flow. These findings can be used in a variety of fields including energy conversion, metal refinery micro-machines and medical treatment.

## Materials and Methods

### Preparation of experimental setup

The experiments were carried out in different electrolytic cells with dimensions of 70 mm × 70 mm × 70 mm and 200 mm × 500 mm × 70 mm. The electrolyte was made of weight 40% potassium hydroxyl mixed with zinc oxide. A stainless steel of 50 mm × 50 mm served as the cathode, a nickel mesh of 50 mm × 50 mm as the anode. A piece of NdFeB magnet of 50 mm × 50 mm was located at the anode side. A cross made of carbon fiber was employed as testing object. In addition, a battery testing instrument (type number CT-4004-10V100A-NTFA) was used for voltage acquisition in experiments.

### Experimental layout

A set of experiments on oxygen bubbles movement was conducted under the condition of the magnet in parallel with the anode. Effects of magnetic induction intensity, electric field and temperature on object movement were also investigated on the basis of the above experimental setup. The experiments on oxygen bubbles movement in Figs 1 and 2 were conducted in the rectangular electrolytic cell with dimension of 250 mm × 500 mm × 70 mm. The rest experiments in Figs 3 and 4 were carried out in the square electrolytic cell with dimension of 70 mm × 70 mm × 70 mm.

## Additional Information

**How to cite this article**: Wang, K. *et al.* Magnetic field induced motion behavior of gas bubbles in liquid. *Sci. Rep.* **6**, 21068; doi: 10.1038/srep21068 (2016).
